# Supplementary material for: Predictors for psychosocial consequences of screening for liver diseases: A data-driven approach
Source: PLoS One. 2025 Apr 29;20(4):e0319488. doi: 10.1371/journal.pone.0319488 (PMC12097750; doi:10.1371/journal.pone.0319488)
Supplement: S1 File — (DOCX) [file pone.0319488.s001.docx]

**Statement on authorship**

The data presented in my manuscript was part of a sub study, which was embedded in the SEAL program. [SEAL – Strukturierte Früh-Erkennung einer Asymptomatischen Leberzirrhose in Rheinland-Pfalz und im Saarland (lebervorsorge.de)](https://www.lebervorsorge.de/seal/web/)

The SEAL program was under the lead of Dr. Nguyen-Tat (PI for the center in Rhineland-Palatinate) and Prof. Dr. Galle (PI for the center in Saarland). All results that refer to the main study, which originates from the medical field, were published by naming all project members.

This sub study was under the lead of Prof. Dr. Farin-Glattacker, who was associated to the project as PI of the sub study, but not of the main study. Therefore, he was not addressed by the ethics committees, since both state medical associations addressed the PI of the responsible centers in the states.

We did not include the PIs (Galle, Nguyen-Tat) as authors, since they were not directly associated with the sub study. We would consider this no good scientific practice to write authors on a paper in this manner. We refer to the Guidelines for Safeguarding Good Research Practice of the DFG. [kodex-gwp-en-data.pdf (dfg.de)](https://www.dfg.de/resource/blob/174052/1a235cb138c77e353789263b8730b1df/kodex-gwp-en-data.pdf):

"The contribution must add to the research content of the publication. What constitutes a genuine and identifiable contribution must be evaluated on a case-by-case basis and depends on the subject area in question. An identifiable, genuine contribution is deemed to exist particularly in instances in which a researcher – in a research-relevant way – takes part in

• the development and conceptual design of the research project, or

• the gathering, collection, acquisition or provision of data, software or sources, or

• the analysis/evaluation or interpretation of data, sources and conclusions drawn from them, or

• the drafting of the manuscript.

If a contribution is not sufficient to justify authorship, the individual’s support may be properly acknowledged in footnotes, a foreword or an acknowledgement."

The two PIs did not fulfill one of the four criteria, and were therefore not listed as authors.
