# Supplementary material for: Predictors for psychosocial consequences of screening for liver diseases: A data-driven approach
Source: PLoS One. 2025 Apr 29;20(4):e0319488. doi: 10.1371/journal.pone.0319488 (PMC12097750; doi:10.1371/journal.pone.0319488)
Supplement: S2 File — (PDF) [file pone.0319488.s002.pdf]

WIE FÜLLE ICH DIESEN FRAGEBOGEN AUS?

Vielen Dank für Ihre Bereitschaft, an unserer Studie teilzunehmen. Bitte beantworten Sie die nachfolgenden Fragen vollständig und gewissenhaft. Mit dieser Umfrage würden wir gerne von Ihnen wissen, wie Sie als Patientin oder Patient im Laufe des SEAL-Programms behandelt wurden und welche Erfahrungen Sie gemacht haben. Die Beantwortung des Fragebogens nimmt etwa 30 Minuten in Anspruch. Alle Angaben werden selbstverständlich vertraulich behandelt und anonym ausgewertet. Bitte schicken Sie den ausgefüllten Fragebogen mithilfe des vorfrankierten Umschlags an uns zurück.

Bitte gehen Sie wie folgt vor:

1. Beantworten Sie die Fragen der Reihe nach.

2. Kreuzen Sie bitte, sofern nicht anders angegeben, für jede Frage nur eine Antwort an.

   Beispiel: Ja………. ☒ Nein……… ☐

3. Bitte setzen Sie pro Zeile nur ein Kreuz in die vorgesehenen Kästchen. Entscheiden Sie sich, wenn nicht anders angegeben, immer nur für eine Antwortmöglichkeit.

   Beispiel:

	Meistens	Ziemlich oft	Manchmal	Selten	Nie
**Richtig:**					
Ich konnte schlecht einschlafen	☐	☒	☐	☐	☐
**Falsch:**					
Ich konnte schlecht einschlafen	☐	☐ X	☐	☐	☐
Ich konnte schlecht einschlafen	☐	☒	☒	☐	☐

4. Überspringen Sie Fragen nur dann, wenn hinter dem von Ihnen angekreuzten Kästchen der Hinweis „Weiter mit Frage…“ steht.

   Beispiel: Ja……… ☐ ▶ Weiter mit Frage…

5. Tragen Sie bitte Zahlen rechtsbündig ein

   Beispiel: Anzahl an Tagen…………………| 0 | 1 | → steht für 1 Tag

6. Falls Sie Antworten korrigieren müssen, nehmen Sie bitte die Korrektur deutlich sichtbar vor.

   Beispiel: Ja………. ☐ Nein………. ■

IHR BESUCH BEIM HAUSARZT

Bei Ihrem Hausarzt / Ihrer Hausärztin wurden erhöhte Leberwerte festgestellt. Wie wurden Sie über Ihren Leberwert informiert?

	**Trifft zu**
Mein/e **Hausarzt/ Hausärztin** machte mich darauf aufmerksam	☐
Ein/e **Facharzt/ Fachärztin** machte mich darauf aufmerksam	☐
**Jemand anderes** machte mich darauf aufmerksam	☐
**Gar nicht.** Das höre ich zum ersten Mal	☐

Bitte geben Sie die Berufsbezeichnung an: ____

Wie wurde Ihnen mitgeteilt, dass Ihre Leberwerte erhöht sind?

	**Trifft zu**
Während einer Behandlungssitzung bei meinem Arzt/ meiner Ärztin	☐
Mein Arzt/ meine Ärztin rief mich persönlich an	☐
Ein/e Praxismitarbeiter/in rief mich persönlich an	☐
Ich erkundigte mich selbst telefonisch bei der Praxis	☐
Per Brief an mich	☐
Gar nicht	☐

Welche möglichen Ursachen wurden Ihnen von Ihrem Hausarzt / Ihrer Hausärztin für diese erhöhten Leberwerte genannt? *(Mehrfachantworten möglich)*

	**Trifft zu**
Alkoholkonsum	☐
Fettreiche Ernährung	☐
Medikamenteneinnahme	☐
Übergewicht (Adipositas)	☐
Leberwerterhöhung in Verbindung mit anderen Erkrankungen, die bei Ihnen vorliegen	☐
Zystische Fibrose	☐
Hepatitis / Virushepatitis	☐
Leberzirrhose	☐
Lebertumor (Krebs)	☐
Entzündungen der Gallenwege / Gallensteine	☐
Andere, und zwar: *(bitte eintragen)* ____________________________________	☐
Keine	☐

Welche Maßnahmen wurden Ihnen von Ihrem Hausarzt / Ihrer Hausärztin genannt, die Sie tun können, um Ihren Leberwert zu senken? *(Mehrfachantworten möglich)*

	Trifft zu
Verzicht auf oder Reduktion von Alkoholkonsum	☐
Ernährungsumstellung in Form einer (fettreduzierten) Diät	☐
Absetzen eines Medikamentes	☐
Sport und Bewegung	☐
Andere, und zwar: *(bitte eintragen)* ______________________________	☐
Keine	☐

Wie zufrieden Sind Sie im Allgemeinen mit diesem Arzt/ dieser Ärztin in Bezug auf…

	Sehr zufrieden	Eher zufrieden	Eher unzufrieden	Sehr unzufrieden
… die Informationen zu den Ursachen erhöhter Leberwerte?	☐	☐	☐	☐
… die Informationen zum Risiko anhaltender hoher Leberwerte?	☐	☐	☐	☐
… die Informationen über mögliche Therapien?	☐	☐	☐	☐
… die Informationen darüber, was Sie selbst auch zur Heilung beitragen können?	☐	☐	☐	☐
… die Verständlichkeit der Informationen?	☐	☐	☐	☐
… die Berücksichtigung aller Behandlungsmöglichkeiten?	☐	☐	☐	☐

Haben Sie Vertrauen zu diesem Arzt/dieser Ärztin?

☐	☐	☐	☐	☐
**Ja, ich habe großes Vertrauen**	**Ja, ich habe eher großes Vertrauen**	**Ich habe eher wenig Vertrauen**	**Nein, ich habe kein Vertrauen**	**Ich kenne den Arzt/die Ärztin nicht lange genug**

Wie schätzen Sie die Qualität der Behandlung durch diesen Arzt/ diese Ärztin im Allgemeinen ein?

☐	☐	☐	☐
**Sehr hoch**	**Eher hoch**	**Eher gering**	**Sehr gering**

Wie zufrieden sind Sie mit diesem Arzt/ dieser Ärztin im Allgemeinen?

☐	☐	☐	☐
**Sehr zufrieden**	**Eher zufrieden**	**Eher unzufrieden**	**Sehr unzufrieden**

© ZAP-Items (Bitzer et al., 1999)

Wurden Sie von Ihrem Hausarzt zur weiteren Abklärung an einen Spezialisten überwiesen?

Ja	☐	▶	Weiter mit der **nächsten Frage**
Nein	☐	▶	Weiter auf **Seite 5**

IHR BESUCH BEIM SPEZIALISTEN

Wie lange müssen/mussten Sie auf einen Termin bei diesem Spezialisten warten?

Insgesamt muss/musste ich |___|___| Wochen warten

Wie zufrieden sind Sie mit der Wartezeit auf einen Termin bei diesem Spezialisten?

☐	☐	☐	☐
**Sehr zufrieden**	**Eher zufrieden**	**Eher unzufrieden**	**Sehr unzufrieden**

Haben Sie den Termin beim Spezialisten bereits wahrgenommen?

Ja	☐	▶	Weiter mit der **nächsten Frage**
Nein	☐	▶	Weiter auf **Seite 5**

Wurden Ihnen von diesem Spezialisten <u>weitere mögliche</u> Ursachen für erhöhte Leberwerte mitgeteilt? *(Mehrfachantworten möglich)*

	**Trifft zu**
Alkoholkonsum	☐
Fettreiche Ernährung	☐
Medikamenteneinnahme	☐
Übergewicht (Adipositas)	☐
Leberwerterhöhung in Verbindung mit anderen Erkrankungen, die bei Ihnen vorliegen	☐
Zystische Fibrose	☐
Hepatitis / Virushepatitis	☐
Leberzirrhose	☐
Lebertumor (Krebs)	☐
Entzündungen der Gallenwege / Gallensteine	☐
Andere, und zwar: *(bitte eintragen)* ______________________________	☐
Keine	☐

Wurden Ihnen von diesem Spezialisten <u>weitere</u> mögliche Maßnahmen zur Senkung Ihrer Leberwerte mitgeteilt? *(Mehrfachantworten möglich)*

	Trifft zu
Verzicht auf oder Reduktion von Alkoholkonsum	☐
Ernährungsumstellung in Form einer (fettreduzierten) Diät	☐
Absetzen eines Medikamentes	☐
Sport und Bewegung	☐
Andere, und zwar: *(bitte eintragen)* ________________________________	☐
Keine	☐

Wie zufrieden Sind Sie im Allgemeinen mit diesem Arzt/ dieser Ärztin in Bezug auf…

	Sehr zufrieden	Eher zufrieden	Eher unzufrieden	Sehr unzufrieden
… die Informationen zu den Ursachen erhöhter Leberwerte?	☐	☐	☐	☐
… die Informationen zum Risiko anhaltender hoher Leberwerte?	☐	☐	☐	☐
… die Informationen über mögliche Therapien?	☐	☐	☐	☐
… die Informationen darüber, was Sie selbst auch zur Heilung beitragen können?	☐	☐	☐	☐
… die Verständlichkeit der Informationen?	☐	☐	☐	☐
… die Berücksichtigung aller Behandlungsmöglichkeiten?	☐	☐	☐	☐

Haben Sie Vertrauen zu diesem Arzt/dieser Ärztin?

☐	☐	☐	☐	☐
**Ja, ich habe großes Vertrauen**	**Ja, ich habe eher großes Vertrauen**	**Ich habe eher wenig Vertrauen**	**Nein, ich habe kein Vertrauen**	**Ich kenne den Arzt/die Ärztin nicht lange genug**

Wie schätzen Sie die Qualität der Behandlung durch diesen Arzt/ diese Ärztin <u>im Allgemeinen</u> ein?

☐	☐	☐	☐
**Sehr hoch**	**Eher hoch**	**Eher gering**	**Sehr gering**

Wie zufrieden sind Sie mit diesem Arzt/ dieser Ärztin im Allgemeinen?

☐	☐	☐	☐
**Sehr zufrieden**	**Eher zufrieden**	**Eher unzufrieden**	**Sehr unzufrieden**

© ZAP-Items (Bitzer et al., 1999)

IHRE EINSTELLUNG

Im Folgenden finden Sie eine Reihe von Feststellungen, mit denen man sich selbst beschreiben kann. Bitte lesen Sie jede Feststellung durch und wählen Sie aus den vier Antworten diejenige aus, die angibt wie Sie sich **jetzt, d.h. in diesem Moment fühlen.** Kreuzen Sie bitte bei jeder Feststellung nur ein Kästchen an.
Es gibt keine richtigen und falschen Antworten. Überlegen Sie bitte nicht lange und denken Sie daran, diejenige Antwort auszuwählen, die Ihren augenblicklichen Gefühlszustand am besten beschreibt.

	Überhaupt nicht	Ein wenig	Ziemlich	Sehr
Ich bin ruhig	☐	☐	☐	☐
Ich fühle mich angespannt	☐	☐	☐	☐
Ich bin aufgeregt	☐	☐	☐	☐
Ich bin entspannt	☐	☐	☐	☐
Ich bin zufrieden	☐	☐	☐	☐
Ich bin besorgt	☐	☐	☐	☐

© STAI-Y-6 - Beltz Test GmbH, Göttingen (Kurzversion nach Marteau et al., 1992)

Bitte kreuzen Sie an, inwiefern Sie folgenden Aussagen zustimmen

	Stimme überhaupt nicht zu	Stimme eher nicht zu	Teils/teils	Stimme eher zu	Stimme voll und ganz zu
Ich bin zufrieden mit den Informationen, die ich über den Ablauf des Screeningverfahrens erhalten habe.	☐	☐	☐	☐	☐
Ich bin zufrieden mit den Informationen, die ich über Risikofaktoren der Lebergesundheit erhalten habe.	☐	☐	☐	☐	☐
Ich bin ausreichend über mögliche Konsequenzen eines erhöhten Leberwert informiert worden	☐	☐	☐	☐	☐
Ich weiß, was ich tun muss, um meinen Leberwert zu senken	☐	☐	☐	☐	☐
Mein erhöhter Leberwert bereitet mir Sorgen	☐	☐	☐	☐	☐
Ich möchte mein Gesundheitsverhalten angesichts meines erhöhten Leberwertes ändern	☐	☐	☐	☐	☐
Ich fühle mich bei meinem behandelnden Hausarzt in guten Händen	☐	☐	☐	☐	☐
Ich fühle mich bei meinem behandelnden Facharzt (falls zutreffend) in guten Händen	☐	☐	☐	☐	☐

KOMMUNIKATIONSBEZOGENE KOMPETENZEN

In diesem Fragebogenteil würden wir von Ihnen gerne wissen, wie Sie sich bisher in Gesprächen mit Ihrem Arzt oder Ihrer Ärztin verhalten haben. Es geht dabei um Ihre persönliche Wahrnehmung und Einschätzung Ihres Verhaltens.

Bitte geben Sie im Folgenden an, wie Sie sich in den Gesprächen mit Ihrem Arzt oder Ihrer Ärztin verhalten haben!

	Trifft voll und ganz zu	Trifft zu	Trifft eher zu	Trifft eher nicht zu	Trifft nicht zu	Trifft überhaupt nicht zu
Falls ich mal anderer Meinung als der Arzt war, habe ich meine Meinung klar geäußert.	☐	☐	☐	☐	☐	☐
Ich habe die Behandlung auch mal kritisch hinterfragt	☐	☐	☐	☐	☐	☐
Ich habe meine eigene Meinung zu Vorschlägen des Arztes geäußert	☐	☐	☐	☐	☐	☐
Falls es Unstimmigkeiten mit dem Arzt gab, habe ich diese klar angesprochen	☐	☐	☐	☐	☐	☐
Ich habe im Gespräch mit dem Arzt auch mal Kritik geäußert	☐	☐	☐	☐	☐	☐
Ich habe vor dem Arzt klar vertreten, was meine Meinung zu der Behandlung ist	☐	☐	☐	☐	☐	☐
Ich habe im Gespräch mit dem Arzt bei passender Gelegenheit Rückfragen gestellt.	☐	☐	☐	☐	☐	☐
Falls ich Zweifel hatte, habe ich diese dem Arzt mitgeteilt	☐	☐	☐	☐	☐	☐
Ich habe dem Arzt Befürchtungen und Ängste bezüglich meiner Behandlung mitgeteilt	☐	☐	☐	☐	☐	☐
Ich habe Fragen zu den Zielen der Behandlung gestellt	☐	☐	☐	☐	☐	☐
Ich bin selbstbewusst aufgetreten	☐	☐	☐	☐	☐	☐
Ich habe dem Arzt Fragen zu Nebenwirkungen der Behandlung gestellt	☐	☐	☐	☐	☐	☐
Ich habe viele Fragen zur Behandlung im Allgemeinen gestellt	☐	☐	☐	☐	☐	☐
Ich habe viele Rückfragen gestellt, um die Erklärungen des Arztes zu verstehen	☐	☐	☐	☐	☐	☐

Ihr Gesundheitszustand

In diesem Fragebogenteil geht es um Ihre Beurteilung Ihres Gesundheitszustandes. Der Bogen ermöglicht es, im Zeitverlauf nachzuvollziehen, wie Sie sich fühlen und wie Sie im Alltag zurechtkommen. Bitte versuchen Sie jede Frage so genau wie möglich zu beantworten.

Wie würden Sie Ihren Gesundheitszustand im Allgemeinen beschreiben?

☐	☐	☐	☐	☐
**Ausgezeichnet**	**Sehr gut**	**Gut**	**Weniger gut**	**Schlecht**

Im Folgenden sind einige Tätigkeiten beschrieben, die Sie vielleicht an einem normalen Tag ausüben. **Sind Sie durch Ihren derzeitigen Gesundheitszustand bei diesen Tätigkeiten eingeschränkt? Wenn ja, wie stark?**	**Ja, stark eingeschränkt**	**Ja, etwas eingeschränkt**	**Nein, überhaupt nicht eingeschränkt**
**Mittelschwere Tätigkeiten**, z.B. einen Tisch verschieben, staubsaugen, kegeln, Golf spielen	☐	☐	☐
**Mehrere** Treppenabsätze steigen	☐	☐	☐

Hatten Sie in der vergangenen Woche aufgrund Ihrer **körperlichen** Gesundheit irgendwelche Schwierigkeiten bei der Arbeit oder anderen alltäglichen Tätigkeiten im Beruf bzw. zu Hause?	**Ja**	**Nein**
Ich habe **weniger geschafft** als ich wollte	☐	☐
Ich konnte **nur bestimmte Dinge** tun	☐	☐

Hatten Sie in der vergangenen Woche aufgrund **seelischer** Probleme irgendwelche Schwierigkeiten bei der Arbeit oder anderen alltäglichen Tätigkeiten im Beruf bzw. zu Hause (z.B. weil Sie sich niedergeschlagen oder ängstlich fühlten)?	**Ja**	**Nein**
Ich habe **weniger geschafft** als ich wollte	☐	☐
Ich konnte nicht so **sorgfältig** wie üblich arbeiten	☐	☐

Inwieweit haben die Schmerzen Sie in der vergangenen Woche bei der Ausübung Ihrer Alltagstätigkeiten zu Hause und im Beruf behindert?

☐	☐	☐	☐	☐
**Überhaupt nicht**	**Ein bisschen**	**Mäßig**	**Ziemlich**	**Sehr**

© SF-12 Hogrefe Verlag GmbH & Co. KG, Göttingen

In diesen Fragen geht es darum, wie Sie sich fühlen und wie es Ihnen in der vergangenen Woche gegangen ist.

Wie oft waren Sie in der vergangenen Woche …

	Immer	Meistens	Ziemlich oft	Manch-mal	Selten	Nie
… ruhig und gelassen?	☐	☐	☐	☐	☐	☐
… voller Energie?	☐	☐	☐	☐	☐	☐
… entmutigt und traurig?	☐	☐	☐	☐	☐	☐

	Immer	Meistens	Manch-mal	Selten	Nie
Wie häufig haben Ihre körperliche Gesundheit oder seelischen Probleme in der vergangenen Woche Ihre Kontakte zu anderen Menschen (Besuche bei Freunden, Verwandten,....) beeinträchtigt?	☐	☐	☐	☐	☐

© SF-12 Hogrefe Verlag GmbH & Co. KG, Göttingen

Wie viele Menschen stehen Ihnen so nahe, dass Sie sich auf sie verlassen können, wenn Sie

IHR UMFELD

ernsthafte Probleme haben?

☐	☐	☐	☐
**Niemand**	**1 oder 2**	**3 bis 5**	**Mehr als 5**

Wie viel Interesse und Anteilnahme zeigen andere Menschen an dem, was Sie tun?

☐	☐	☐	☐	☐
**Sehr viel**	**Viel**	**Weder viel noch wenig**	**Wenig**	**Kein Interesse und Anteilnahme**

Wie einfach ist es für Sie, praktische Hilfe von Nachbarn zu erhalten, wenn Sie diese benötigen?

☐	☐	☐	☐	☐
**Sehr einfach**	**Einfach**	**Möglich**	**Schwierig**	**Sehr schwierig**

© Oslo Social Support Scale (Dalgard et al., 2006)

Gesundheitliche Probleme

Im folgenden Teil finden Sie eine Liste verschiedener gesundheitlicher Probleme.

Wir möchten gerne wissen, welche der aufgeführten gesundheitlichen Probleme Sie haben.

Bitte kreuzen Sie an, ob Sie das betreffende gesundheitliche Problem haben und wenn ja, wie schwer Sie das Problem einschätzen (leicht, mittel oder schwer).

Bitte bearbeiten Sie jede Zeile!

	**Haben Sie das folgende gesundheitliche Problem? Wenn ja, wie schwer schätzen Sie es ein?**			
	**nein**	**ja**		
		**leicht**	**mittel**	**schwer**
Bluthochdruck	☐	☐	☐	☐
Herzinfarkt (in der Vergangenheit)	☐	☐	☐	☐
Durchblutungsstörung/ Gefäßkrankheiten	☐	☐	☐	☐
Erkrankung der Atmungsorgane	☐	☐	☐	☐
Magen-Darm-Erkrankung	☐	☐	☐	☐
Erkrankung der Leber/Galle	☐	☐	☐	☐
Nierenerkrankung	☐	☐	☐	☐
Diabetes (Zuckerkrankheit)	☐	☐	☐	☐
Erkrankung des Muskel-Skelett-Systems (z.B. chron. Rückenschmerzen, Gelenkerkrankungen)	☐	☐	☐	☐
Neurologische Erkrankung (z.B. Schlaganfall, Multiple Sklerose)	☐	☐	☐	☐
Krebs	☐	☐	☐	☐
Niedergeschlagenheit, Ängstlichkeit	☐	☐	☐	☐

© KoMo-Score Patient (Glattacker, Meixner, Farin & Jäckel, 2007)

Ihr Umgang mit medizinischen Informationen

In welchem Maß haben Sie ganz allgemein in den Gesprächen mit Ärzten, Therapeuten oder Pflegekräften Schwierigkeiten …

	Keine	Geringe	Mäßige	Große	Sehr große
medizinische Informationen, die Ihnen mitgeteilt werden, zu verstehen.	☐	☐	☐	☐	☐
medizinische Ratschläge, die Sie erhalten, zu Hause im Alltag anzuwenden.	☐	☐	☐	☐	☐
zu verstehen, was die medizinischen Informationen für Ihre eigene Erkrankung bedeuten.	☐	☐	☐	☐	☐
die vielen Informationen zu verstehen.	☐	☐	☐	☐	☐
das Wesentliche vom Unwesentlichen zu unterscheiden.	☐	☐	☐	☐	☐
eigene Probleme und Themen einzubringen.	☐	☐	☐	☐	☐
Ihre Fragen zu diskutieren.	☐	☐	☐	☐	☐
Ihre eigenen Erwartungen und Wünsche bezüglich der Behandlung mitzuteilen.	☐	☐	☐	☐	☐
auch sehr persönliche Fragen zu Ihrer Erkrankung zu stellen.	☐	☐	☐	☐	☐

© HELP-Fragebogen (Farin, Nagl & Ulrich, 2013)

IHRE ERFAHRUNGEN MIT DEM LEBERSCREENING

Wir möchten mehr über Ihre Erfahrungen mit dem SEAL-Lebervorsorgeprogramm erfahren. Bitte beantworten Sie die folgenden Fragen so gut Sie können.

Wie oft haben Sie in der vergangenen Woche folgende Dinge aufgrund von Gedanken und Gefühlen über Lebererkrankungen erlebt?

	Überhaupt nicht	Selten	Manchmal	Oft
Ich hatte Schwierigkeiten zu schlafen	☐	☐	☐	☐
Ich hatte einen veränderten Appetit	☐	☐	☐	☐
Ich war unglücklich oder niedergeschlagen	☐	☐	☐	☐
Ich war verängstigt oder beunruhigt	☐	☐	☐	☐
Ich war nervös oder angespannt	☐	☐	☐	☐
Ich habe mich belastet gefühlt	☐	☐	☐	☐
Ich habe Dinge vor mir nahestehenden Personen verheimlicht	☐	☐	☐	☐
Ich habe meine Laune an anderen Menschen ausgelassen	☐	☐	☐	☐
Ich habe mich von mir nahestehenden Personen zurückgezogen	☐	☐	☐	☐
Ich hatte Schwierigkeiten damit, die Dinge zu Hause zu erledigen, die ich normalerweise erledige	☐	☐	☐	☐
Ich hatte Schwierigkeiten, meine Arbeit zu erledigen oder anderen Verpflichtungen nachzukommen	☐	☐	☐	☐
Ich habe mir Sorgen um die Zukunft gemacht	☐	☐	☐	☐

Alles in allem, würden Sie sagen, dass Ihre Erfahrung mit dem SEAL-Lebervorsorgeprogramm Folgendes bei Ihnen ausgelöst hat?

	Überhaupt nicht	Etwas	Ziemlich	Sehr
Ich fühle mich sicher, dass ich keinen Leberschaden habe	☐	☐	☐	☐
Ich fühle mich entspannter	☐	☐	☐	☐
Meine Beziehung zu meinen Freunden oder Angehörigen hat sich verbessert	☐	☐	☐	☐
Ich fühle mich mehr dazu in der Lage, die Dinge zu unternehmen, die ich früher unternommen habe	☐	☐	☐	☐
Ich fühle mich mehr dazu in der Lage, meinen Haushalts- oder Arbeitsverpflichtungen nachzukommen	☐	☐	☐	☐
Ich blicke hoffnungsvoller in die Zukunft	☐	☐	☐	☐
Ich habe weniger Angst vor Lebererkrankungen	☐	☐	☐	☐
Ich komme mit Menschen in meinem Umfeld besser klar	☐	☐	☐	☐
Ich kann besser schlafen	☐	☐	☐	☐
Ich fühle mich insgesamt wohler	☐	☐	☐	☐

PCQ (Cockburn et al., 1992)

Wie sehr hat sich durch das Leberscreening im Rahmen des Checkups Ihre Gesundheit bis heute im Vergleich zu vor dem Checkup verändert?

	Nicht gehabt	Stark verschlechtert	Etwas verschlechtert	Gleich geblieben	Etwas verbessert	Stark verbessert
Erschöpfung, Müdigkeit	☐	☐	☐	☐	☐	☐
Niedergeschlagenheit, Angst	☐	☐	☐	☐	☐	☐
Nervosität, Unruhe	☐	☐	☐	☐	☐	☐
Angespanntheit, Aufgeregtheit	☐	☐	☐	☐	☐	☐
Verletzbarkeit, Verletzlichkeit	☐	☐	☐	☐	☐	☐
Hoffnungslosigkeit	☐	☐	☐	☐	☐	☐
Schwierigkeiten, anderen Menschen zu vertrauen	☐	☐	☐	☐	☐	☐
Schwierigkeiten, Bekanntschaften oder Freundschaften aufrechtzuerhalten	☐	☐	☐	☐	☐	☐

QS-Reha-Bogen DRV

IHRE EINSTELLUNG ZU SCREENINGS

In diesem Fragebogenteil interessieren wir uns für Ihre Pläne und Gedanken zum Thema Lebergesundheit in der Zukunft.

Bitte überlegen Sie, inwiefern Ihre Lebervorsorge in diesem Jahr Ihre zukünftige Inanspruchnahme von Lebervorsorgeprogrammen verändern könnte.
Welche Aussage passt am besten zu Ihrer aktuellen Meinung?

	Trifft zu
Es ist jetzt weniger wahrscheinlich als früher, dass ich in der Zukunft wieder an einem Lebervorsorgeprogramm teilnehmen werde.	☐
Meine Bereitschaft, an Lebervorsorgeprogrammen teilzunehmen wird sich nicht ändern	☐
Es ist jetzt wahrscheinlicher als früher, dass ich in der Zukunft wieder an einem Lebervorsorgeprogramm teilnehmen werde.	☐
Keine Angabe/ weiß nicht	☐

Planen Sie innerhalb der nächsten 2 Jahre wieder an einem Lebervorsorgeprogramm teilzunehmen?

☐	☐	☐
**Ja**	**Nein**	**Weiß nicht/ noch nicht entschieden**

Persönliche Angaben

Bitte geben Sie Ihr Geschlecht an.

☐	☐	☐
**Männlich**	**Weiblich**	**Anderes / Keine Angabe**

In welchem Jahr sind Sie geboren?

|__|__|__|__| (Jahr)

Wie viele Personen leben insgesamt in Ihrem Haushalt? (Sie einbezogen)

Anzahl an Personen: |__|__|__|__|

Haben Sie eine/n festen Lebenspartner?

	**Trifft zu**
Ja, wir leben in einem gemeinsamen Haushalt	☐
Ja, wir leben in getrennten Haushalten	☐
Nein	☐
Keine Angabe	☐

Welche der Kategorien auf dieser Liste beschreibt am besten, wo Sie wohnen?

	**Trifft zu**
Großstadt	☐
Rand oder Vororte einer Großstadt	☐
Mittel- oder Kleinstadt	☐
Ländliches Dorf	☐
Allein stehendes Haus auf dem Land	☐

Welchen höchsten allgemeinbildenden Abschluss haben Sie?

	**Trifft zu**
(noch) keinen Abschluss	☐
Haupt-/Volksschulabschluss	☐
Realschulabschluss (mittlere Reife) oder Abschluss der Polytechnischen Oberschule	☐
Fachhochschulreife	☐
Allgemeine oder fachgebundene Hochschulreife (Abitur)	☐

Welchen höchsten berufsbildenden Abschluss haben Sie?

	Trifft zu
(noch) keinen Abschluss	☐
Lehre (Berufsausbildung im dualen System)	☐
Fachschule (Meister, Techniker)	☐
Berufs- oder Fachakademie	☐
Fachhochschulabschluss	☐
Universität / wissenschaftliche Hochschule	☐
Promotion	☐

Was trifft <u>überwiegend</u> auf Sie zu?

	Trifft zu
Ich bin erwerbstätig (inkl. Altersteilzeit und Elternzeit)	☐
Schüler/-in; Auszubildende/r; Studierende/r	☐
Rentner/-in; Pensionär/-in	☐
Hausfrau; Hausmann	☐
Arbeitslos	☐
Dauerhaft arbeitsunfähig	☐

Stellen Sie sich vor, diese Leiter zeigt an, wo Menschen in ihrem sozialen Umfeld stehen.

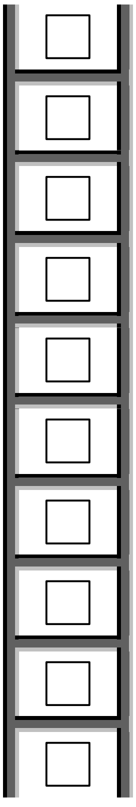

10
9
8
7
6
5
4
3
2
1

Menschen unterscheiden sich darin, was sie unter sozialem Umfeld verstehen. Bitte definieren Sie soziales Umfeld so, wie es für Sie am sinnvollsten ist.

An der Spitze der Leiter sind Menschen, die in ihrem sozialen Umfeld den höchsten Stand haben. Am unteren Ende der Leiter stehen Menschen, die in ihrem sozialen Umfeld den niedrigsten Stand haben.

Je höher Sie auf der Leiter stehen, desto ähnlicher sind Sie den Menschen am oberen Ende der Leiter. Je niedriger Sie auf der Leiter stehen, desto ähnlicher sind Sie den Menschen am unteren Ende.

Wo würden Sie sich auf der Leiter platzieren?

Bitte kreuzen Sie an, auf welcher Leitersprosse Sie zum gegenwärtigen Zeitpunkt im Vergleich zu anderen Menschen in Ihrem sozialen Umfeld stehen.

© MacArthur Scale (Adler et al., 2000)

Vielen Dank für Ihre Teilnahme!

Bitte schicken Sie diesen Fragebogen mithilfe des vorfrankierten Umschlags an uns zurück.
