# Supplementary material for: Predictors for psychosocial consequences of screening for liver diseases: A data-driven approach
Source: PLoS One. 2025 Apr 29;20(4):e0319488. doi: 10.1371/journal.pone.0319488 (PMC12097750; doi:10.1371/journal.pone.0319488)
Supplement: Attachment — Submitted filename: Response to reviewers.docx [file pone.0319488.s005.docx]

## Response to reviewers

# Dear Dr. Campbell

Dear Reviewers

We thank you for considering our manuscript and we are grateful for the valuable feedback. We carefully went through all the points raised during the review process. Please find our answers point by point below. We hope that the changes made will improve the quality and readability of our manuscript.

Journal Requirements:

1. When submitting your revision, we need you to address these additional requirements.

Please ensure that your manuscript meets PLOS ONE's style requirements, including those for file naming. The PLOS ONE style templates can be found at 
https://journals.plos.org/plosone/s/file?id=wjVg/PLOSOne_formatting_sample_main_body.pdf and 
https://journals.plos.org/plosone/s/file?id=ba62/PLOSOne_formatting_sample_title_authors_affiliations.pdf

2. Thank you for stating the following financial disclosure: "funded by the Innovation Fund of Federal Joint Committee of Germany, provided by the Deutsches Zentrum für Luft- und Raumfahrt (Funding ID: 01NVF16026)".

Please state what role the funders took in the study.  If the funders had no role, please state: "The funders had no role in study design, data collection and analysis, decision to publish, or preparation of the manuscript."

If this statement is not correct you must amend it as needed.

Please include this amended Role of Funder statement in your cover letter; we will change the online submission form on your behalf.

3. Your ethics statement should only appear in the Methods section of your manuscript. If your ethics statement is written in any section besides the Methods, please move it to the Methods section and delete it from any other section. Please ensure that your ethics statement is included in your manuscript, as the ethics statement entered into the online submission form will not be published alongside your manuscript.

4. Please include captions for your Supporting Information files at the end of your manuscript, and update any in-text citations to match accordingly. Please see our Supporting Information guidelines for more information: http://journals.plos.org/plosone/s/supporting-information.

🡪 Thank you for this remark. We made some corrections related to the style of the document to meet the style requirements provided by PLOS. We updated the cover letter and included the funding statement. We moved the Ethics Statement to the Methods section. We further added a section "Supporting information" which includes the captions for the four files.

Reviewer #1: ##How much time had passed since the subjects had been screened and then responded to the mailed surveys? Any concerns about recall difficulties or bias?

##I would be more convinced by a study which used a smaller sample but who more closely and prospectively tied their investigation of screening effects to the screening procedures themselves while utilizing mixed methods (qualitative interviews regarding patients' experiences with liver screening would be a valuable addition to the literature). The vast majority of these patients were screened negative and one would wonder what kind of negative psychology that would yield in them. If authors REALLY wanted to query psychological responses to liver disease screening, they should employ methods which investigate patient populations with more liver risks are present (i.e., more test subjects receiving positive screening results) and investigating patient populations where leading cirrhosis diagnoses (i.e., alcohol-related cirrhosis, Met-ALD, MASLD) are more likely to be present. As examples, conducting cirrhosis screening in patient populations seeking care in addiction or obesity clinics. They could also investigate the psychological impact of screening patients in the general population for excessive alcohol use--a key factor in liver damage and cirrhosis development in many countries. In my experience, discussion of excessive alcohol use and discussion of hazardous behaviors leading to liver disease (excessive drinking, overeating) ROUTINELY cause psychological distress in patients. I would also be interested in more granular detail about HOW patients received their screening data--who delivered the information and what kind of interaction took place as the results and implications were discussed.

🡪Thank you for raising this point. We agree that a mixed methods approach is always the best way to gather a deeper understanding of the research field. In fact, we also conducted a qualitative sub-study, which showed some detailed insights into the psychosocial consequences in line with the screening. We added some more information on the results of this sub-study in the introduction part to draw a more complete picture. In this manuscript, however, we focus less on describing the consequences of screening and more on identifying contextual factors that may be associated with it. The last point related to the communication and interaction of the results is very important! We share this opinion and therefore we also gathered data on this. We added a paragraph in the discussion section, which highlights the importance on how results are communicated.

##In sum, I wasn't surprised by the test results that in a sample comprised of largely persons receiving negative screening results about their livers and later asked to reflect on the psychosocial effects of this, that there was not much distress reported. I am certainly glad that screening for cirrhosis doesn't elicit distress--it is an important endeavor. Yet, my years of experience caring for at-risk liver patients, and reflections on what I've seen, suggests that the reality is likely to be quite different than this study's findings. For most people, receiving unflattering objective data about their liver health tied to health behaviors which people already tend to misrepresent to their physicians (diet, alcohol consumption) is often a distressing experience. In fact, it is this unpleasant psychological experience that liver clinicians and their psychosocial colleagues tend to harness therapeutically in order to nudge patients into making changes in their lifestyle to improve their liver health. Part of me reads this literature about the psychological effects of medical screening the way we might consider literature about the pain perceptions of patients undergoing IV placement--isn't it inevitable and perfectly normal that they will be uncomfortable to some degree?

🡪Thank you for this statement. Our study tried to get insights in the amount of distress that the screening could produce and we are also glad that this amount is quite low in a broad population. However, there are some special cases, especially those with high risk for liver cirrhosis, which might be the complicated ones you are referring to from your years of experience. Patients might tend to bias their reflections, especially when cognitive dissonance occurs. When they receive unflattering objective data that indicates a damaging behavior, which the patient is in charge of, they might feel compromised and try to whitewash reality. Since the at-risk liver patients are luckily quite low in the broad population of screening, we had a only a small sample size in our study which limits the analysis potential for this specific group. However, we think that recall bias is an important aspect that also needs to be discussed and therefore we added this point in the limitations section.

##With so many psychometric instruments being used and without ready access to the items they contain and which domains were queried, it was difficult to parse what factors were influencing what. The domains were already rather nebulous as to how they were defined (e.g., what are the discrete postulated psychosocial consequences of medical screening?).

🡪 Thank you for this comment. We now added the original questionnaires we used as supplementary material to give the readers access to the items and domains measured. The PCQ measures three components: psychological, emotional and physical dysfunction in the context of screening.

Reviewer #2: I read with interest the manuscript entitled "Predictors for psychosocial consequences of screening for liver diseases: A data-driven approach". I especially enjoyed the systematic approach in this study.

I have a few concerns and comments.

The study seems to lack data about the specific phase in which each participant was within the screening process. This apparently leads to the inability to study the consequences of false positive screening results.
Response rate was 9-12%, and apparently only 21 individuals with a positive screening result were included.

Another limitation is the assessment of a mixture of short- and long-term consequences of screening, since participants were at different stages of the screening process.

Large part of the text on pages 17-18 would be more appropriately located in the discussion

Was the screening result (pos vs neg) considered in the multivariable models? This should be clarified.

🡪Thank you for reviewing our article and for the critical points you remarked. Yes, unfortunately we do not have information on the specific phase the patients were within the screening process. As consequence, we had a mixture of short- and long-term consequences of screening. We consider this as major limitation and hope that we made that clear in the manuscript. Within the nature of the screening procedure, the positive screening rate was quite low and thus, we also had a very low sample that was positively screened. We extended the discussion part to make sure that our critical reflection on this topic is understandable.

Regarding your question of the screening result: yes, this was modeled as a potential predictor, but the LASSO approach did not categorize this variable as important predictor. We now also integrated a paragraph on this in the discussion section.

We hope we addressed all the points raised by the reviewers adequately and finally want to thank you all again for your critical input which helped to improve the quality of this manuscript.
