# Supplementary material for: Predictors for psychosocial consequences of screening for liver diseases: A data-driven approach
Source: PLoS One. 2025 Apr 29;20(4):e0319488. doi: 10.1371/journal.pone.0319488 (PMC12097750; doi:10.1371/journal.pone.0319488)
Supplement: Attachment — Submitted filename: Rebuttal_letter_new.docx [file pone.0319488.s007.docx]

Dear Editor

Thank you for reviewing our article carefully. We appreciate your feedback and made some changes in our manuscript. Below, please find our answers to your recommendations point by point in green. We hope that the changes made contribute to a better comprehension of our work.

*The manuscript entitled, “Predictors for psychosocial consequences of screening for liver diseases: A data-driven approach” investigated the predictors to explain variance in post-screening psychosocial consequences in the context of a liver screening for cirrhosis and fibrosis. They also try to establish the protective and negative impact variables. The manuscript is written in good English and I thought the flow of the manuscript needs to be improved. Most importantly, the study design and recruitment of study participants. This research should clarify the ethics and consent of study populations. Therefore, the current form of the manuscript needs to go through the revision. I appreciate the authors improving the manuscript, and to resubmit. I want to specify some major comments for improving the manuscript.
Abstract:
It would be appreciated if the authors showed the statistical values (for example, beta coefficient with 95% CIs) in the results sections. The conclusion part is too long in comparison to the results. It should be reduced by highlighting the specifications.*

- Thank you for this feedback. We added the beta coefficients and a sentence adressing the CIs. From our point of view, writing down all CIs would limit readability of the abstract. We hope the suggested solution meets the request appropriately. We further erased text in the conclusion part to shorten it down in comparison to the results section.

*Introduction:
The study's main aim was to identify the psychosocial consequences of liver diseases, specifically liver cirrhosis and fibrosis. Although the study background states a lot of things, unfortunately, it misses out on the importance of psychosocial consequences of liver diseases that are well studied till now. It would be appreciated if the authors would consider the aforementioned point. Additionally, the objectives of the study are stated in a question sentence. It is better to avoid the question sentences even with numbering.*

- Thank you for raising this point. We added a paragraph that focuses on the psychosocial and economic consequences of liver diseases and added relevant literature on that. We further rephrased our research questions into aims instead of questions.

*Methodology:
I want the authors to state the advantages of LASSO regression over other conventional regression analyses. Is it appropriate only for variable selection? Why did the authors conduct 100 bootstrapping? Did you conduct sensitivity analyses? If so, please specify.
Please state the participant's rights and confidentiality statement.*

- Thank you for requesting more information on the methodology. We added some details and hope these illustrate the rationale for the methodic approach. We did not conduct any sensitivity analyses, however the 10-fold cross-validation for determination of lambda could be considered as such. We extended the text in the ethics statement section to clarify that we only processed data when written informed consent was given. If requested, we can also provide the patient information and consent document.

*Results:
The overall response rate is too low. Does not it have the effect of analysis bias and overall findings?*

- We agree that this limitation should be discussed. We added elaboration on the response rate in the discussion section.

*Discussion and conclusion:
Strengths and limitations are merged in one section. The author should clarify the strengths and limitations separately.*

- Thank you for this comment. We separated the discussion section in summary, limitations and strengths. We hope this supports readability of our manuscript.

*The overall discussion is not up to the mark, it should be more specific in the context of the findings.*

- *Thank you. We added a new paragraph, which places the results more firmly in the existing literature. We hope this contributes to a more specific discussion.*
